# Raspberry Leaves and Extracts-Molecular Mechanism of Action and Its Effectiveness on Human Cervical Ripening and the Induction of Labor

**DOI:** 10.3390/nu15143206

**Published:** 2023-07-19

**Authors:** Maciej W. Socha, Wojciech Flis, Mateusz Wartęga, Monika Szambelan, Miłosz Pietrus, Anita Kazdepka-Ziemińska

**Affiliations:** 1 Department of Perinatology, Gynecology and Gynecologic Oncology, Faculty of Health Sciences, Collegium Medicum in Bydgoszcz, Nicolaus Copernicus University, Łukasiewicza 1, 85-821 Bydgoszcz, Poland; 2 Department of Obstetrics and Gynecology, St. Adalbert’s Hospital in Gdańsk, Copernicus Healthcare Entity, Jana Pawła II 50, 80-462 Gdańsk, Poland; 3 Department of Pathophysiology, Faculty of Pharmacy, Collegium Medicum in Bydgoszcz, Nicolaus Copernicus University, M. Curie-Skłodowskiej 9, 85-094 Bydgoszcz, Poland; 4 Department of Pharmacology and Therapeutics, Faculty of Medicine, Collegium Medicum in Bydgoszcz, Nicolaus Copernicus University, M. Curie-Skłodowskiej 9, 85-094 Bydgoszcz, Poland; 5 Department of Gynecology and Oncology, Jagiellonian University Medical College, 31-501 Kraków, Poland

**Keywords:** obstetrics, pregnancy, labor induction, cervical ripening, nutrition, supplement, raspberry leaves, perinatal metabolism, perinatal metabolomics

## Abstract

The gestational period is an incredibly stressful time for a pregnant woman. Pregnant patients constantly seek effective and reliable compounds in order to achieve a healthy labor. Nowadays, increasing numbers of women use herbal preparations and supplements during pregnancy. One of the most popular and most frequently chosen herbs during pregnancy is the raspberry leaf (*Rubus idaeus*). Raspberry extracts are allegedly associated with a positive effect on childbirth through the induction of uterine contractions, acceleration of the cervical ripening, and shortening of childbirth. The history of the consumption of raspberry leaves throughout pregnancy is vast. This review shows the current status of the use of raspberry leaves in pregnancy, emphasizing the effect on the cervix, and the safety profile of this herb. The majority of women apply raspberry leaves during pregnancy to induce and ease labor. However, it has not been possible to determine the exact effect of using raspberry extracts on the course of childbirth and the perinatal period. Additionally, it is unclear whether this herb has only positive effects. The currently available data indicate a weak effect of raspberry leaf extracts on labor induction and, at the same time, their possible negative impact on cervical ripening.

## 1. Introduction

The gestational period is a stressful time for a pregnant woman. Pregnancy involves many physiological adaptations that may challenge a woman’s health. In recent years, there has been a significant improvement in the awareness of women (and often their partners) regarding the changes that occur during pregnancy, pregnancy-associated health risks, nutritional awareness, and the overall course of pregnancy [1,2,3]. This is mainly due to the large number of available educational programs, and the wide availability of the internet [4]. Currently, due to the growing health awareness among pregnant women, an increasing number of women are consuming healthy foods during pregnancy. Therefore, a higher number of women follow an appropriate diet, and use nutritional supplements during pregnancy. Recent studies show that a significant percentage of the surveyed women reach for supplementary preparations during pregnancy. In particular, the use of herbal products has been recorded among pregnant patients, and the majority of the population, to promote health in recent decades [5,6]. It is estimated that the rate of use of herbal preparations during pregnancy is approximately 50% worldwide [7]. One of the most popular and most frequently chosen herbs during pregnancy is the raspberry leaf (*Rubus idaeus*). The awareness of pregnant women regarding the use of raspberry leaves during pregnancy seems to be constantly growing, and ranges from 7% to 56%, depending on the country [8]. Women reach for this specimen in the form of tablets, tea, or a tincture. Red raspberry leaves have been ingested as a uterotonic agent and “general pregnancy” tea for the last two centuries [8,9]. The use of the raspberry herb is believed to play a role in the prevention of miscarriage, the alleviating of discomfort in the prodromal labor stage, the prevention of overdue pregnancy, the alleviation of morning nausea, the induction of labor, or an enhanced cervical ripening. Additionally, red raspberry leaf is often administered to alleviate labor-associated pain. Whilst the history of raspberry leaf ingestion in the gestational period is long, the available research does not contribute much to the evidence base, especially concerning its mechanism of action, efficacy, effectiveness, or possible side effects [10,11,12,13,14]. Moreover, there are very limited data regarding the biological properties of the substances in raspberry leaves.

This review aims to determine raspberry leaves’ possible mechanism of action, effectiveness, and side effects in pregnancy and childbirth, particularly emphasizing the impact on labor induction and cervical ripening.

## 2. Red Raspberry

According to their botanical definition, berries are seed fleshy fruits formed from a single ovary that does not contain a drupel, and their core is created with a triple layer. The leading representative of the Rubus species (belonging to the Rosacaea family) is *Rubus idaeus*, also known as the red raspberry or European red raspberry. *Rubus idaeus* is widely cultivated in Asia, Europe, and North America. Red raspberries are one of the most consumed berries worldwide; recent figures suggest that the annual production of berries is over 800k metric tons. The leading producers of these fruits are Poland, Mexico, Russia, and Serbia. [15,16]. Other edible berries (Rubus spp.) include blueberries, cranberries, blackberries, gooseberries, black and red currants, and strawberries.

Raspberry fruits are available as a freeze-dried, frozen, or fresh commodity. They are very widely used in food manufacturing, for jams, wines, and juices. Additionally, their processing generates a large amount of different byproducts, such as leaves and stems, which can be recycled and embodied into new food, pharmaceutical, or cosmetic products. Raspberry leaves are used as a diet supplement, mainly in the formulation of teas or chocolate, ameliorating their flavor and nutritional properties [17,18,19,20,21,22].

The main components of the dry matter of berries are fiber and monosaccharides (such as glucose and fructose). They also contain a large number of inorganic ions (zinc, calcium, copper, manganese, magnesium, selenium, phosphorus, and potassium).

They are rich in vitamins such as vitamins C, A, and E, which are known for their antioxidant activity. The inorganic composition is strongly influenced by the place where the plant is grown, and the method of cultivation [23,24]. Besides inorganic compounds, raspberries contain many bioactive molecules, such as polyphenols, tocopherols, carotenoids, and fatty acids. The group of polyphenols includes flavonoids (anthocyanins, kaempferol, catechin), phenolic acids (mainly ellagic acid), and stilbenes (such as resveratrol). Anthocyanins—a group of flavan-3-ols such as malvidin, cyanidin, petunidin, delphinidin, pelargonidin, and peonidin—manifest a strong antioxidant activity, and are responsible for the color of the fruits of these plants [20,24,25,26,27]. The compound with the highest concentration in raspberry leaves is ellagic acid. Red raspberries contain ellagic acid, which occurs in three forms: as free ellagic acid, ellagic acid glycoside, and as ellagitannins, in which hexahydroxydiphenic acid is combined into esters with sugar. Ellagic acid makes up approximately 50% of the dry mass of all phenols in raspberries, and shows a strong antiproliferative effect [24]. Another group of compounds found in berries comprises saturated and polyunsaturated fatty acids, such as myristic acid or linoleic acid. In addition, small amounts of quercetin and its derivatives, kaempferol, tocopherol, ellagitannins, and various catechins, can be found in raspberries [25,26].

The high number of polyphenols (flavonoids, phenolic acids, and stilbenes) may strongly contribute to natural antioxidant production. All of the above substances demonstrate potent antioxidant and antiproliferative effects. It is believed that the benefits of consuming extracts of these plants are the result of a strong antioxidant and antiproliferative effect. Additionally, an increased consumption of the phenolic compounds contained in raspberries may be associated with a decrease in the incidence of diseases associated with oxidative stress [27,28,29,30]. 

Apart from the biological components contained in the dry matter of the berries’ leaves, raspberry seed oil is a known source of valuable bioactive components [22,31]. Seed oil is extracted via a hydro-distillation, maceration, or cold-pressing technique. The quality and concentration of the substances highly depend on the type of raspberry, the cultivation region, and the oil extraction technique. The raspberry seed oil contains a high amount of polyunsaturated fatty acids, tocopherols, polyphenols, and p-anisidine [31]. This unique content of various bioactive molecules makes the oil a source of ingredients for food, or for the cosmetics and pharmaceutics industries. We believe that raspberry seed oil may significantly impact human health. However, further extensive research is required in this exciting area.

## 3. Possible Impact on Labor and Cervical Ripening

Currently, no studies clearly demonstrate that the consumption of raspberry leaves has an unequivocal effect on the molecular and biochemical pathways of the labor period. In this section, we will discuss the potential effect of raspberry leaves on cervical remodeling and childbirth pathways.

**Figure 1:**
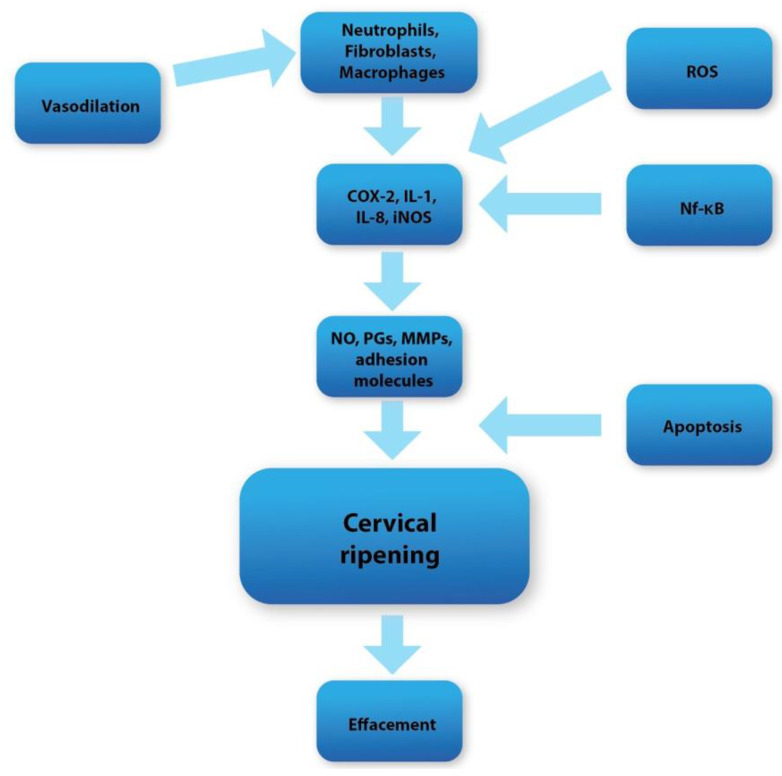
Diagram showing overall cervical ripening molecular pathways.

Human parturition can be divided into two major phases. During the first phase, cervical ripening occurs in the last days of gestation, or is iatrogenically triggered during the labor induction protocol. Cervical ripening can be broadly defined as a series of biochemical and molecular complex pathways accompanied by oxidative stress, inflammation, and apoptosis, whose activation leads to the alteration of the spatial conformation of the extracellular matrix of the cervical tissue [32]. As a result, there is an increased water influx, the decomposition of collagen fibers, and the development of a local inflammatory reaction [33,34,35]. The effect of these processes (which are regulated on many levels) is a significant increment in the softening and susceptibility of the uterine cervix (Figure 1). Through these changes, the cervical tissue loses its physical integrity and becomes more prone to dilation [34,36,37]. The second phase, called active labor, can be characterized by regular uterine systolic activity, which leads to the expulsion of the fetus from the uterus. The above stages are strongly related, and any disturbances can lead to the failure of proper vaginal delivery. 

## 4. Raspberries vs. Apoptosis and Reactive Oxygen Species (ROS)

The period of human pregnancy is programmed in advance. One of the elements of the cervical ripening pathway is apoptosis, which is most pronounced in the perinatal period [38]. Studies show that in a rat cervix, a great increase in the apoptosis of cervical muscle cells can be observed at full-term pregnancy [39]. 

Apoptosis is an essential process in cell auto-destruction, which is pivotal for supporting correct homeostasis, and occurs in every living organism. During this process (due to numerous biochemical pathways), the fragmentation of the nucleus, chromatin condensation, membrane blebbing, and cell shrinking can be observed. Subsequently, pro-apoptotic bodies are formed, which are enzymatically digested by macrophages [40,41].

Apoptosis is mediated by the caspase–endoprotease enzymes family, which can split peptide bonds in their substrate [41]. Caspases are primarily produced as zymogens that require enzymatic cleavage for activation. Their substrates include cell structural proteins, DNA, and cell cycle regulatory molecules. Caspases can affect actin polymerization, leading to cytoskeleton destruction. Another caspase substrate is the mitochondrial electron transport chain. These enzymes can directly interrupt the electron transport chain by cleaving the p75 subunit of complex I in the mitochondria [42]. This, in turn, uncouples oxidative phosphorylation and is, hence, a critical function of ATP synthesis. As well as directly impacting the cell structure, caspases can participate in inflammation by forming inflammasomes. An activated and assembled inflammasome directly enhances the synthesis of inflammatory cytokines, such as interleukin-1 (IL-1), a key player in the inflammatory response, and pivotal proinflammatory cytokines in cervical ripening [43].

A variety of pathways can trigger apoptosis. The mitochondrial apoptosis pathway is under the regulation of the permeability of the inner mitochondrial membrane, which is impermeable under physiological conditions. In certain circumstances (such as excessive reactive oxygen species production), the permeability of the mitochondrial internal membrane significantly increases, which leads to an influx of protons to the mitochondrial matrix, uncoupling oxidative phosphorylation, and causing the osmotic oedema of the mitochondrial matrix. Subsequently, this results in the laceration of the mitochondrial membrane [44]. Then, cytochrome c is expulsed into the cytosol, intensely stimulating apoptosome formation. Active apoptosomes can enhance caspase expression [45]. 

Another apoptotic pathway—the endoplasmic pathway—is associated with Ca^2+^ ions. The accumulation of calcium ions in the mitochondrial matrix may affect the permeability of the mitochondrial membrane, resulting in osmotic swelling. This leads to the disruption of the mitochondrial membrane, and the release of cytochrome c into the cytosol [46]. 

Finally, the extrinsic apoptosis pathway is maintained by the tumor necrosis factor receptors (TNF-Rs), a TNF receptor superfamily chained with the cell surrounding membrane. The stimulation of these receptors directly activates caspases into their active enzymatic forms [44,47].

Reactive oxygen species (ROS) are free radicals which contain spare electrons. Unpaired electrons provide them with a great reactivity. While correct homeostasis is maintained, small amounts of ROS are formed, which actively regulate cell signaling, acting as second messengers via the opening of ion channels or the activation of protein kinases, protein formations, and apoptosis [48]. Physiologically, ROS production is under the strict control of the antioxidant systems. However, a sharp fluctuation between pro- and antioxidant properties can lead to oxidative stress (OS), which can be defined as an excessive ROS production [49,50]. Undoubtedly, labor and pregnancy are strongly related to increased reactive oxygen and nitrogen species, resulting in oxidative stress.

Reactive oxygen species can, directly and indirectly, participate in every apoptosis pathway that occurs during cervical ripening [38,44]. As a result of excessive ROS formation in the cell, the permeability of the internal mitochondrial membrane increases. This causes an increased influx of protons into the mitochondrial matrix, which leads to the burst of the mitochondrial membrane, and the expulsion of cytochrome c into the cytosol [51]. Additionally, reactive oxygen species can directly oxidize cardiolipin molecules, which triggers the liberation of cytochrome c into the cytosol. Reactive oxygen species may activate p53 N-terminal kinase and/or c-Jun (JNK), which enhance the activation and synthesis of the proapoptotic Bcl-2 proteins (located in the mitochondrial outer membrane) that inhibit the anti-apoptotic pathways [38,44,47,48,49,50,51,52,53]. Finally, ROS may directly trigger the outburst the of Ca^2+^ stored in the endoplasmic reticulum, which may activate the endoplasmic apoptosis pathway. 

Research has revealed that treating cervical cells with oxidative stress inducers (e.g., cigarette smoke extract) activated ROS synthesis, promoting cervical cell cycle arrest and inducing cell apoptosis [38]. In summary, oxidative stress contributes to apoptosis during cervical ripening, and both these processes appear to be conjugated [38,44]. 

The substances contained in raspberries (e.g., tocopherols, flavonoids, ellagic acid, zinc, anthocyanins, and vitamins C and E) demonstrate strong antioxidant properties [48,54,55,56,57,58,59]. Considering the high antioxidant bioactivity of the substances contained in raspberries, it seems reasonable to assume that these substances may have some impact on the processes occurring in the cervical tissue during its ripening.

The antioxidant effects of these substances are direct and indirect. Their direct effect mainly consists of scavenging ROS via the electron transfer of free radicals, and by inhibiting specific oxidases [54], whereas their indirect antioxidant effect increases the concentration of oxidative enzymes (such as or glutathione peroxidase or superoxide dismutase), ROS synthesis inhibition, and lipid peroxidation inhibition [60,61,62]. Considering the above, we believe that the bioactive antioxidant substances contained in raspberries can significantly participate in reducing the concentration of ROS (or increasing their scavenging) in the cervical tissue, which may negatively affect its ripening in the perinatal period. However, the influence of these substances on the apoptosis process should not be overlooked. Studies have unequivocally shown that the bioactive antioxidative molecules in raspberries (ellagic acid, flavonoids, and anthocyanins) have an antiproliferative effect, and can directly stimulate cell apoptosis and autophagy [63]. Ellagic acid (belonging to the group of phenolic acids), the concentration of which remains at a high level in raspberry dry mass, may have a proapoptotic effect, by inhibiting cyclin-dependent kinases [64,65]. Cyclin-dependent kinases (CDKs), which are serine/threonine protein kinases, are crucial players in regulating the cell cycle. The activity of CDKs is influenced by cyclins and specific CDK inhibitors (CKIs). The CDK4 and CDK6 isoforms are the main components of the cell cycle pathway for driving proper cell division. CDK6 kinase actively participates in glucose metabolism via the phosphorylation and inhibition of two pivotal enzymes of the glycolytic pathway: pyruvate kinase and 6-phosphofructokinase (6-PFK). This, in turn, leads to the redirection of the intermediary substances of the glycolytic pathway into the serine and pentose phosphate pathways. The inhibition of CDK6 decreases the activity of those pathways, leading to the depletion of the antioxidants NADPH and glutathione peroxidase. This, in turn, increases the concentration of ROS, which may directly activate apoptotic pathways [66]. Ellagic acid can both inhibit the expression of CDK6 in the cell, and directly inhibit its regulatory activity. Therefore, by inhibiting CDK6, ellagic acid can increase the concentration of ROS in the cell, which may increase the permeability of the inner mitochondrial membrane, and oxidize cardiolipin, which activates the apoptosis pathways.

Moreover, studies have also revealed that using raspberry extract stimulates the apoptosis of cervical cancer cells [67]. Furthermore, flavonoids (contained in blueberries) also show proapoptotic properties in breast and lung cancer cells [68,69].

Considering all of the above, we believe that raspberries may have a significant relationship with the biochemical pathways occurring in the cervical stroma during its maturation. Theoretically, it seems that the consumption of raspberries in the perinatal period may somewhat affect cervical ripening. The bioactive components of blueberries may negatively affect cervical ripening, by reducing the available pool of ROS. On the other hand, by stimulating apoptosis, they can also enhance the maturation of the cervix. However, considering the two possible pathways of influence on the cervix, it is necessary to determine which of these processes may be dominant. We believe that these issues require further meticulous research.

## 5. Raspberries vs. Inflammation in Cervical Tissue

One of the main components of the cervical remodeling process is the local inflammation that develops in the cervical tissue [32]. The mediators maintaining the proper course of inflammation are crucial factors regulating the cervical ripening process. In the perinatal period, local vasodilation occurs, which causes an increase in the vascular permeability. Subsequently, an increased inflammatory cell influx and water inflow can be noticed. As a result, there is a significant increase in the concentration of inflammatory cells in the cervical extracellular matrix (ECM), such as macrophages, mast cells, and neutrophils, at full term [37,70,71]. These cells are responsible for the secretion of nitric oxide (NO), adhesion molecules, prostaglandins (PGs), metalloproteinases (MMPs), and proinflammatory cytokines. These substances are among the most crucial factors maintaining the proper course of maturation in the cervix [71]. 

Interleukin-8 (IL-8), secreted mainly by fibroblasts, is a potent neutrophil chemotactic agent that regulates cervical maturation. By increasing the vascular permeability, it has a chemotactic effect on neutrophils. In addition, IL-8 stimulates neutrophils to secrete their MMPs (which can cleave collagen covalent bonds), and enhances the expression of interleukin-1 (IL-1) [72,73].

Research has unequivocally shown that the vaginal administration of IL-1 suppositories causes a significant increase in the compliance of the cervix [74]. Therefore, it seems that one of the most substantial factors affecting cervical maturation is IL-1. IL-1 affects the cervix mainly by increasing the expression of COX-2 (responsible for synthesizing prostaglandins), while downregulating PG dehydrogenase (PGDG), leading to an increased concentration of active PGs in the cervical stroma. In addition, IL-1 increases the secretion of MMPs by neutrophils and fibroblasts, and increases the synthesis of other proinflammatory cytokines, such as IL-6, IL-8, and TNF [75,76].

Prostaglandins, which are derivatives of arachidonic acid, have a significant effect on both the cervix and the uterine corpus [32,77]. In addition to being able to generate uterine contractions, they actively participate in cervical remodeling. They affect the female reproductive system through a family of membrane-coupled EP1–EP4 receptors [78]. PGs directly affect the composition of the cervical ECM, by enhancing the synthesis of glycosaminoglycans (GAGs) by fibroblasts and stromal cells. In addition, they increase the total water content, and reduce the amount of collagen in the extracellular matrix. PGs can directly enhance the activity of MMPs, which subsequently decompose collagen fibers. Prostaglandins also strongly affect the influx of inflammatory cells to the cervical stroma. They intensify the synthesis of IL-8, the strongest chemotactic factor of neutrophils. Secondly, they increase the expression of adhesion molecules in endothelial cells, such as ICAM-1, further enhancing the influx of leukocytes [79,80,81]. The prostaglandins remain closely supervised by other factors regulating the course of cervical ripening. Proinflammatory cytokines can both stimulate the synthesis of PGs, and upregulate the expression of prostaglandin receptors (EP1–EP4) in the cervical tissue [82,83].

Another crucial factor that strongly impacts the cervical ECM composition is nitric oxide (NO). According to studies, the vaginal application of NO donors can effectively induce cervical ripening [84].

Nitric oxide is derived from L-arginine under the influence of specific nitric oxide synthases (NOSs) [85,86]. The predominant nitric oxide synthase in cervical tissue is inducible NOS (iNOS) [87]. Its activity is not constitutive, but only induced by specific factors, such as lipopolysaccharides or cytokines. Neutrophils and macrophages are the main sources of NO in the cervical ECM, as they can express significant amounts of iNOS at full-term pregnancy [87]. Nitric oxide may exhibit its effects on the surrounding tissues both via the direct and indirect pathway. The direct effect is manifested by upregulating the activity of guanylate cyclase, which converts GTP to cGMP. Subsequently, cGMP acts as a second messenger [87,88]. The indirect effect includes nitration or oxidation, which leads to the alteration of the protein structure, and the formation of reactive nitrogen species (RNS). The effect of NO on the cervical ECM is mainly manifested through an enhancement in the expression of metalloproteinases (mainly MMP-9 and MMP-2), which degrade collagen covalent bonds. Additionally, NO participates in inflammatory cell influx by stimulating the secretion of IL-8 (a powerful neutrophil chemotactic agent), and inducing local vasodilatation [89].

NO is a potent PG synthesis inducer. Nitric oxide can directly induce COX-2 expression, significantly increasing the PG production in the cervical stroma [90,91,92]. 

In addition to the main performers in the cervical maturation process, there is also a group of overriding factors whose role is to modulate the proper course of cervical ripening. Nuclear factor-kappaB (Nf-kB) is a superfamily of transcription factors, such as p52, cRel, p52, and RelA/p65 and RelB, which, after activation, gain the ability to translocate to the nucleus, where they exhibit transcriptional activity, leading to the enhanced transcription of the genes encoding factors that regulate cervical ripening [93,94,95]. Nf-kB remains under the heavy influence of hormonal factors (e.g., progesterone and glucocorticoids) and inflammatory factors [96]. After translocating to the nucleus, Nf-kB enhances the expression of inflammatory mediators, such as IL-1, IL-8, IL-6, COX-2, and iNOS, which are key regulators of cervical ripening [95,96,97]. Interestingly, a regulatory metabolic loop appears to exist in the cervix. As mentioned, Nf-kB may enhance the expression of iNOS and COX-2, which leads to an increase in the synthesis of PGs and NO. However, it transpires that both NO and PGs can also positively affect Nf-kB expression. Nitric oxide can generate large amounts of peroxynitrite (ONOO^−^), which is a reactive form of nitrogen. Peroxynitrite shows a high affinity to Nf-kB, leading to the constant activation of Nf-kB. Subsequently, Nf-kB nuclear activity is constantly maintained, and thus the continuous transcription of Nf-kB-dependent genes is ensured [98]. In turn, prostaglandins can affect Nf-kB through their receptors. PGs act, among others, through the EP4 receptor (which works via protein kinase A and cAMP). Studies have shown that an increased cAMP concentration stimulates protein kinase A, which activates Nf-kB. This results in Nf-kB’s translocation to the nucleus, and an enhancement in the transcription of the genes encoding proinflammatory cytokines, NO, and PGs [99].

Oxidative stress (along with an excessive ROS production) occurs at the maternal–fetal unit from early pregnancy, gradually increasing at full term. Apart from playing a significant role in apoptosis, ROS and RNS play a substantial role in ripening [100]. As mentioned earlier, the neutrophil concentration increases during cervical changes. Thanks to their peroxidases (mainly NADPH oxidases), neutrophils produce a significant amount of ROS. Reactive oxygen species (in addition to inducing a proapoptotic effect) can affect Nf-kB activity via the phosphorylation of Nf-kB-activating kinase (NIK). Subsequently, Nf-kB becomes activated, leading to an enhancement in the transcription of specific genes coding for proinflammatory cytokines, PGs, and NO [97,101,102,103]. In addition, reactive oxygen species may also exert their indirect effects on the cervix through p38MAPK [32,101,104]. Mitogen-activated protein kinases are proline-directed threonine and serine protein kinases, which are intensely expressed in macrophages [104]. As mentioned earlier, the concentration of macrophages significantly increases during the cervical remodeling process [32]. The p38 family of proteins (subtypes of MAPK) is a group of kinases activated by factors such as cytokines and TLR ligands. It is believed that p38MAPK participates in the macrophage-mediated inflammation process. The phosphorylation of p38 kinase leads to its activation and subsequent translocation to the nucleus. In turn, active p38MAPK can activate the transcription of proinflammatory genes [105]. The inflammatory involvement of p38MAPK is manifested through the increased expression of inflammatory cytokines, COX-2 and NO. Additionally, p38MAPK can directly upregulate the expression of MMPs (especially MMP-9), which participate in collagen breakdown. Finally, p38MAPK enhances the leukocytic influx by enhancing the expression of VCAM-1 (vascular cell adhesion molecule-1) [104,105,106,107,108]. Proinflammatory cytokines and ROS influence the activity of p38MAPK. Excess ROS and an increased concentration of cytokines (mainly IL-1) lead to the direct activation of p38MAPK, which translates into a multiplication of the expression of proinflammatory factors [104].

Through an understanding of the main biochemical and molecular pathways occurring during cervical ripening, it should be possible to answer the question of the relationship between these pathways and the substances contained in raspberries. The antioxidants contained in raspberries are able to scavenge ROS directly, and reduce their formation. This class of anthocyanins (whose concentration in raspberries is high) has been known for some time, especially regarding its antioxidative effectiveness [109,110]. Anthocyanins are closely related to cellular oxidative stress levels. The transcription factor nuclear-factor-erythroid-2-related factor 2 (Nrf2) is sensitive to the redox status. During physiological conditions, Nrf2 is inactivated and bonded to the Kelch-like ECH-associated protein 1 (Keap1) in the cell cytosol [111]. When the concentration of ROS increases, and oxidative stress increases, the Nrf2 disrupts from Keap1, enabling the translocation of the Nrf2 into the nucleus. Then, the activated Nrf2 binds to antioxidant-responsive elements (AREs), regulating the transcription of the genes coding the enzymes crucial for antioxidant defense, such as heme oxygenase-1 (HO-1) or NAD(P)H quinone oxidoreductase-1 (NQO-1) [112,113,114]. According to various studies, anthocyanins may directly stimulate the Nrf2/ARE signaling pathway, leading to a significant reduction in the concentration of ROS [115,116]. We believe that by affecting the Nrf2/ARE pathway, the anthocyanins contained in raspberries can significantly contribute to reducing the impact of ROS on the biochemical pathways that occur during cervical ripening. As a result, they may substantially affect the histological changes occurring in the cervical stroma at full term. In addition, numerous studies also indicate that the ellagitannins (e.g., ellagic acid) contained in raspberries also significantly affect antioxidant properties, leading to a reduction in the amount of ROS generated, and an increase in the activity of antioxidant enzymes (such as superoxide dismutase and catalase) [117,118,119]. Considering the above, we believe that the bioactive substances contained in raspberries, through their active indirect and direct influence on the redox status in the cell, can negatively affect the remodeling of the cervix. However, we suggest that further research is required to conclusively confirm the effect of these substances on the cervical tissue.

In addition to their effect on cell redox status, the substances contained in raspberries can also modulate the course of the local inflammatory reaction. Referring to recent research, using raspberry extract on non-alcoholic fatty liver disease (NAFLD) in rat hepatic cells significantly reduced the concentration of the Nf-κB transcription factor [24,120]. This may suggest that raspberry extract, by reducing the concentration of Nf-κB, may lead to a decreased production of proinflammatory cytokines and COX-2, which can undoubtedly ameliorate the inflammatory response. We believe that this phenomenon can be explained by the inactivation of molecules that regulate the activity of Nf-κB. NF-kB is a family of factors which, in its inactive form, forms a complex with the NF-kB inhibitor proteins IκBα, IκBβ, and IκBε. These three inhibitor isoforms can bind and inactivate NF-κB subunits [120,121]. Inhibitors are regulated by IκB kinase (IKK). IKK can phosphorylate IκBα, which leads to its degradation, allowing Nf-κB to translocate to the nucleus [103]. According to studies, anthocyanins (contained in raspberry extract) can directly reduce the phosphorylation of IκBα and IKK, leading to the inhibition of the activity of Nf-κB [122]. Subsequently, this leads to a decrease in the synthesis of NO, COX-2, and proinflammatory cytokines. 

Additionally, anthocyanins and flavonoids may directly enhance anti-inflammatory properties, leading to the suppression of the inflammatory response. Studies have shown that the use of raspberry extracts or raspberry seed oil (which is rich in phenolic compounds) may cause a significant decrease in the macrophage-mediated inflammatory reaction by directly reducing the expression and activity of PGE2 and COX-2 [21,122,123,124]. Additionally, raspberry extracts are associated with a marked diminution in the mRNA levels of iNOS and the concentration of nitric oxide [122,124]. Finally, flavonoids and phenolic acids can significantly reduce the expression and concentration of the proinflammatory cytokines-IL-1 and IL-6 [122,125,126,127,128].

**Figure 2:**
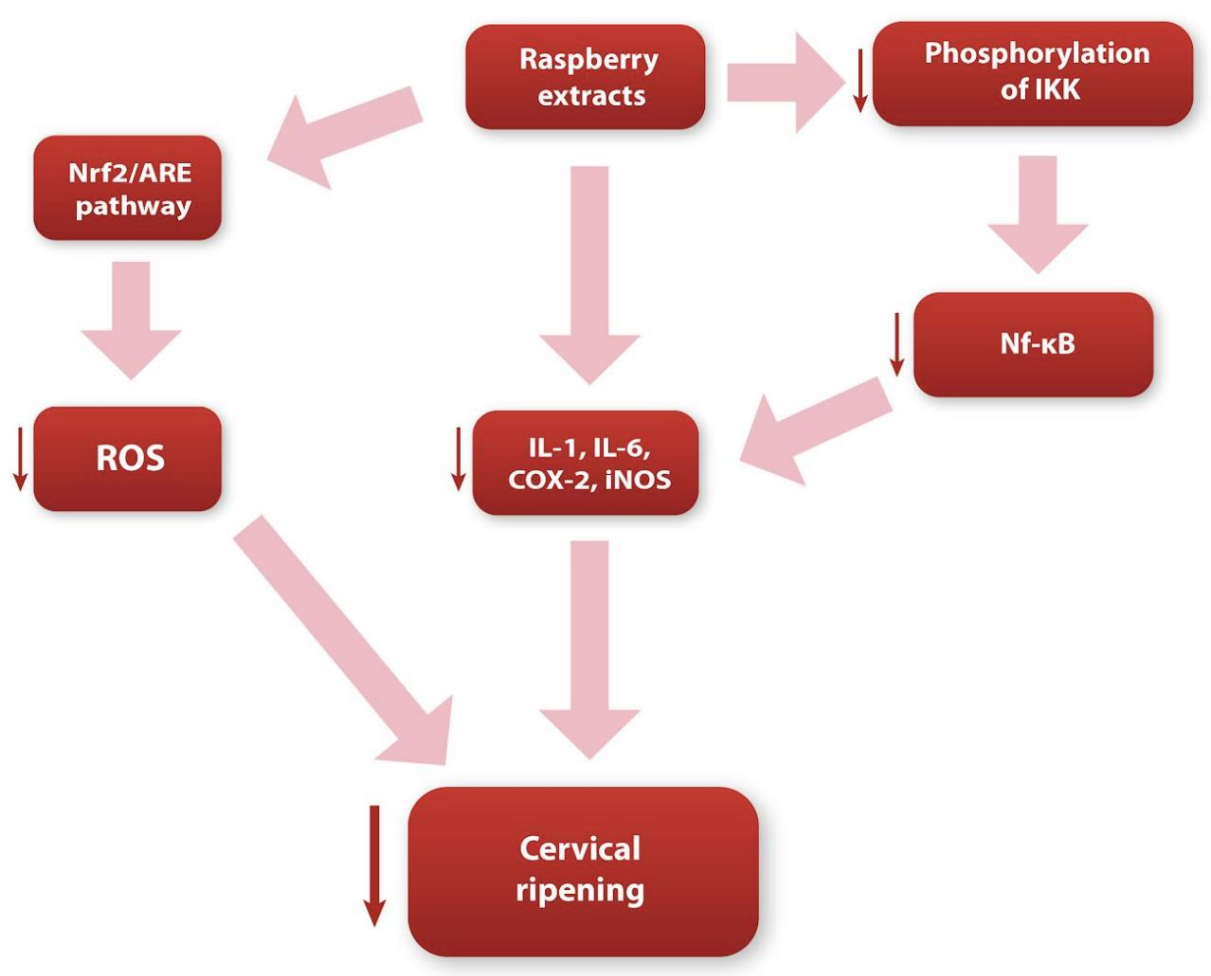
Diagram showing possible ways in which raspberry extracts influence the cervical ripening process.

Considering all of the above, we believe that the bioactive, antioxidant substances contained in raspberries (e.g., anthocyanins and flavonoids) significantly contribute to inhibiting the inflammatory response and reducing reactive oxygen species concentrations (Figure 2). Bearing in mind the extremely active participation of the local inflammatory reaction and oxidative stress in the cervical ripening pathways, we believe that the consumption of raspberries may significantly inhibit the biochemical pathways that occur during cervical remodeling. However, we also think that this topic requires additional research, to clearly determine the effect of raspberry extracts on the cervical tissue.

## 6. Discussion

The search for effective and reliable compounds to aid pregnancy and labor has received great attention worldwide, but has achieved only partial success. Pregnant women have been ingesting herbal products for centuries. This often causes pregnant women to self-medicate, including the use of herbal supplements and herbal-based medicines. According to research, the use of herbal products has significantly increased worldwide among pregnant patients. The use of herbs is subject to variability, depending on the patient’s cultural traditions, socio-economic status, and geographical region [129,130]. One of the herbs that have recently gained an increased popularity is *Rubus idaeus*, the red raspberry. Raspberry extract (typically ingested as a daily tea) is allegedly reported to be effective in promoting cervical ripening, and inducing labor. However, in reality, the impact of raspberries on pregnancy and parturition is not well understood. Moreover, little is known about the safety and efficacy of the ingestion of raspberries on the human female reproductive system. To the best of our knowledge, this is the first extensive review that summarizes the possible effects of raspberries on both labor induction and cervical ripening, with additional emphasis on their potential impact on biochemical and molecular pathways.

There are several studies evaluating the contractile effect of using raspberry extracts. However, the presented results are very ambiguous and questionable. Research in murine models showed that raspberry extracts had a volatile impact on uterine systolic activity, depending on the gestational age, preexisting contractile activity, and herbal preparation. Moreover, even if the contraction effect was achieved, it was negligible. This research does not support the hypothesis that raspberry extract may augment labor by directly affecting the uterine contractility [131]. Additionally, another study highlighted that raspberry extraction showed a relaxation effect [132]. It is also worth remembering that the studies conducted so far may not be entirely reliable. The results may vary depending on the method of extraction, herbal preparation, type of tissue examined, baseline uterine muscle tonus, and gestational age [133,134]. One of the mechanisms in which raspberry extracts could affect the contractile activity of the uterus is their effect on nitric oxide. In addition to the apparent effect on the ripening of the cervix, NO, through its ability to relax blood vessels, preserves a low resistance to flow in the umbilical and uterine vessels. Therefore, it maintains the proper perfusion of the materno–fetal surface [85]. Due to the action of progesterone, cervical nitric oxide production is suppressed during pregnancy, whilst uterine NO production is increased [84,134]. Due to the action of raspberry compounds (such as flavonoids), there is a noticeable decrease in the NO concentration. Theoretically, the reduction in the NO concentration in the uterine muscle may contribute to abolishing the uterine quiescence provided by NO. This, in turn, may increase the susceptibility of the uterine muscle to contraction stimuli.

In conclusion, we believe that raspberry extracts do not present a contractile effect on the uterine muscle and, even if this effect occurs, it is not very highly expressed, and is insignificant, so it will not lead to the release of regular uterine contractions. However, we believe that further thorough research is required to consider all variables, to demonstrate the effect of raspberry extracts on uterine contractility.

One of the studies we reviewed suggested that the consumption of raspberries may positively influence the course of labor by reducing the rate of cesarean section, and of operative deliveries using obstetric forceps or an obstetric vacuum [135]. However, the results of this research should be interpreted with caution, due to the very small study group and the study’s retrospective nature.

In addition to considering the possible mechanisms of action in pregnancy and delivery, we should also consider the possible side effects of consuming raspberries. According to recent studies, the consumption of raspberry extracts may be associated with the occurrence of hypoglycemic episodes, which is a dangerous complication, especially in women with gestational diabetes [136]. Moreover, raspberry extracts may interact with other medications. Referring to the research, raspberry leaf extracts may lead to the clinically significant downregulation and inhibition of intestinal CYP3A4 (an element of the cytochrome P450 superfamily), significantly altering the absorption and pharmacokinetics of any medications taken [137]. Finally, the substances contained in raspberries can have an impact on coagulation. Studies have shown that ellagic acid (which occurs in a high concentration in raspberries) has a significant hyper-coagulant effect; the use of ellagic acid has been associated with shortening clotting times (PT and APPT) and increasing thrombin activity [138]. Pregnancy is a state of physiological hyper-coagulability. The consumption of substances that may have an additional pro-coagulant effect is particularly dangerous, due to the relevant increase in the risk of the occurrence of thromboembolic complications, such as deep vein thrombosis. An increasing number of pregnant women are reaching for herbal preparations, especially raspberry extracts. In this study, we highlight potential herb–drug interactions and possible side effects, to draw clinicians’ attention to the need to collect a thorough history regarding the use of all preparations by patients. We draw attention to this because, currently, there are no guidelines specifying the dosage of these preparations, and their possible side effects. This can lead to disastrous consequences for the mother and her unborn child.

Our review shows that consuming raspberry extracts can negatively affect the cervical ripening. However, our considerations are purely theoretical, and show the possible impact of the given substances contained in raspberries on the cervix. We would like to point out that it is not known in what concentration the raspberry extracts may exhibit the described effect. Moreover, little is known about whether the substances contained in raspberry extracts can affect the cervix tissue itself. There are currently no dosing guidelines available for these supplements. In conclusion, we believe thorough research should be carried out, to accurately assess the effect of individual ingredients on the cervix, and check at what concentration of given substances they will achieve their effect.

Many pregnant women ingest raspberry extracts during pregnancy to facilitate an easier and more harmonious labor. However, as this review has shown, there is currently no evidence suggesting that women should take these preparations during pregnancy. The evidence based on the studies conducted so far on the use of raspberry extracts during pregnancy shows that they do not provide any benefit. Moreover, scant evidence suggests that raspberry leaves have a disadvantageous effect, especially on the cervical ripening pathways. Our review shows that the consumption of raspberry extracts could translate into decreased dynamics, or even the inhibition of the cervical ripening process, which could undoubtedly translate into a more tumultuous and traumatic childbirth course. However, it should be remembered that these considerations are purely theoretical, and concern only possible interactions between the biochemical pathways in the cervix and how they are influenced by the substances contained in raspberries. We believe that these considerations are an excellent starting point for further studies that could assess the effect of raspberries on the apoptosis process and oxidative stress that occur during cervical ripening.

In addition, we have also shown that the consumption of raspberry extracts may be associated with unexpected and not-thoroughly-researched side effects. Due to the popularity of using raspberry extracts during pregnancy, there is a constant drive to fully understand the impact of these substances on the course of pregnancy and labor. Therefore, we believe that further profound and precise research is required to determine the validity of this information.

## Data Availability

Data sharing is not applicable.
